# The complete mitochondrial genome of *Bradysia impatiens* (Diptera: Sciaridae)

**DOI:** 10.1080/23802359.2022.2080594

**Published:** 2022-06-20

**Authors:** Yang Wang, Caixia Liu, Qingyun Wang, Hong Wu, Junhao Huang

**Affiliations:** National Joint Local Engineering Laboratory for High-Efficient Preparation of Biopesticide, Zhejiang A&F University, Hangzhou, Zhejiang, China

**Keywords:** *Bradysia*, fungus gnat, mitogenome, Sciaroidea, species evolution

## Abstract

*Bradysia impatiens* (Johannsen, 1912) is an important pest of various mushrooms and plant seedlings, while it also damages plants as vectors transmitting pathogens. The complete *B*. *impatiens* mitogenome is 16,479 bp in length, with an A + T content of 80.6%. The mitogenome had three overlapped regions; the longest overlap is 7 bp. Phylogenetic analysis reveals that *B*. *impatiens* is closely related to *Bradysia amoena*. The study provided a basis for future studies of species divergence in *Bradysia*.

Many *Bradysia* species are discovered as agricultural pests, as their larvae feed on mycelia, plant roots, and decayed plant tissues. Several species in the *B*. *tilicola* group, including *B*. *impatiens* (Johannsen, 1912), *B*. *ocellaris* (Comstock, 1882), and *B*. *odoriphaga* (Zhang, 1985), are worldwide pests that damage economically important plant seedlings and edible fungi (Sueyoshi and Yoshimatsu 2019; Yang et al. 2019). *Bradysia impatiens* has a short life cycle of about 20 days, with 12–15 generations produced annually (Xi et al. 2021). The species has a broad range of plant hosts, including lily, spring onion, broad bean and cucumber, in China, Brazil and Australia, etc. (Broadley et al. 2018). It can transmit the plant pathogens *Verticillium albo-atrum*, *Botrytis cinerea*, and *Pythium aphanidermatum* through its feeding and excretion activities (Gou et al. 2020; Xi et al. 2021). These studies mainly focused on the taxonomy and biological control of *Bradysia*. As the genus with the greatest species richness in the family Sciaridae, *Bradysia* was regarded as polyphyletic (Shin et al. 2013), and the phylogenetic status of *B*. *impatiens* remains unclear. Here, we assembled the complete mitochondrial genome of the species to analyze its phylogenetic relationships with other Sciaridae species, and to provide a basis for future speciation research.

Insect material was collected in Songxian County, He’nan Province, China (30°53′7″N, 112°10′6″E) in May 2020. The specimen was deposited at Zhejiang A&F University, China under the voucher number WQY013 (https://www.zafu.edu.cn/, Junhao Huang, huangjh@zafu.edu.cn). Genomic DNA was exacted from the thorax of an adult male using a Qiagen® DNeasy Blood & Tissue kit (Hilden, Germany). The genomic DNA libraries (350 bp) was generated using Illumina HiSeq 4000 platform. Adapter sequences and low-quality reads (base pairs ≤40 or ‘N’ ≥10) were trimmed using Trimmomatic 0.36 (Bolger et al. 2014). Approximately, 4,218 Mb of clean data was obtained. The mitogenome was assembled by the SPAdes v3.14.0 and IDBA-UD v1.1.3 (Bankevich et al. 2012; Peng et al. 2012), and then was annotated using Geneious Prime v11.0.9. Maximum likelihood (ML) trees were constructed using IQ-TREE v1.6.12 (Trifinopoulos et al. 2016). The study has been reviewed by the Experimental Animal Ethics Committee of Zhejiang A&F University. Ethical approval is not required for the sample collection and study of this insect species.

The total mitogenome length (GenBank accession. no. MZ202360) is 16,479 bp, with an A + T content of 80.6%. The circular mitogenome contains 37 genes, 22 transfer RNAs (tRNAs), 2 ribosomal RNAs (rRNAs) and 13 protein-coding genes (PCGs) (Figure S1). The J-strand of the *B. impatiens* mitogenome is composed of 25 genes, whereas the N-strand hosts the remaining 12 genes (Table S1). There are three overlapping regions (*atp8*-*atp6*; *trnlF*-*nad5*; *nad4*-*nad4l*); the longest overlap is 7 bp. The total length of the 13 PCGs is 11,232 bp. All genes are in the same locations and strands with the species from the same subfamily Megalosphyinae (*Dolichosciara megumiae*, *Bradysia amoena* and *Bradysia* sp.) (Miao et al. 2020). Meanwhile, the positions and orientation of protein-coding genes are similar to previously sequenced fungus gnats (Wang et al. 2021).

**Figure 1.**
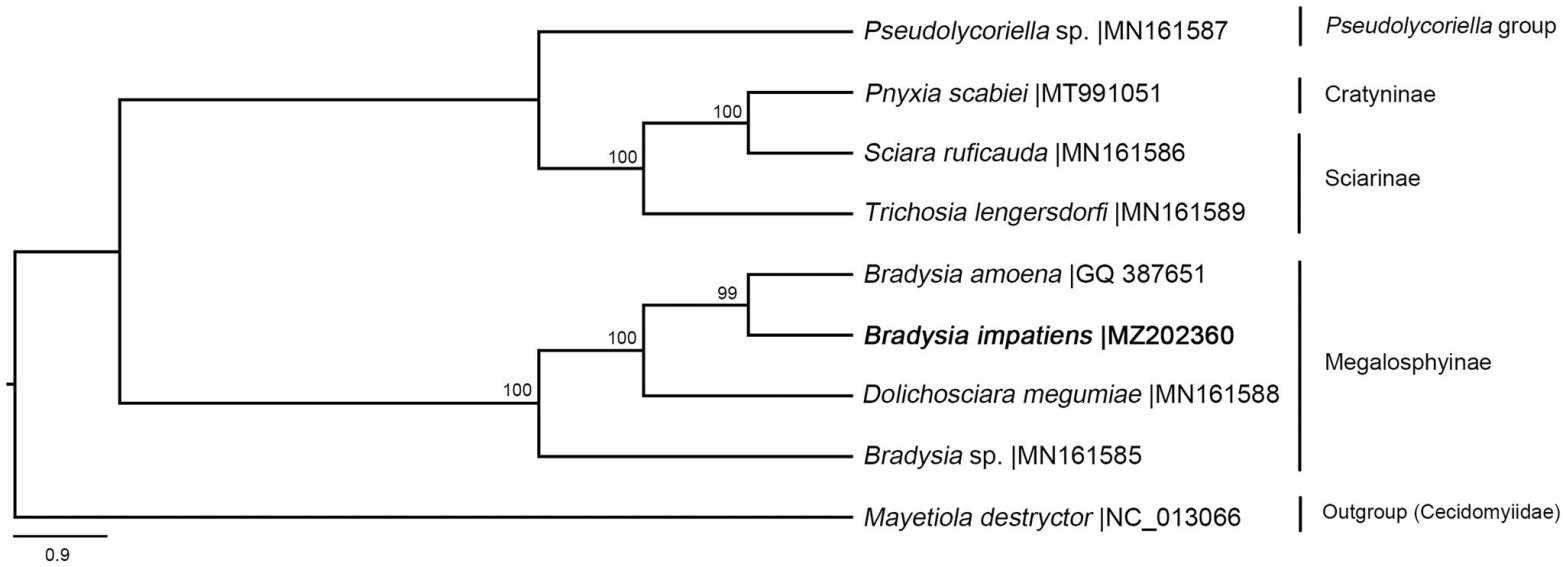
Phylogenetic tree of eight species of Sciaridae. The number on branches refers to the degree of confidence (%). Clades are labeled with names of subfamily or genus group of the family.

In the 13 PCGs, five have ATA start codons (*cox2*, *atp8*, *cox3*, *nad6* and *cob*), while the other start codons are ATG (*nad2*, *cox1* and *atp6*), AAT (*nad5*, *nad4l* and *nad1*), ATT (*nad3*), CAT (*nad4*), and ATT (*nad1*). The stop codons of the PCGs include eight TAA (*nad2*, *cox1*, *cox2*, *atp8*, *atp6*, *cox3*, *nad3* and *cob*), two CTA (*nad5* and *nad1*), two TTA (*nad4* and *nad4l*), and one TAG (*nad3*). Codon usage of the *B. impatiens* mitogenome is presented in Table S2. The mostly frequently codons (TTA-Leu, ATT-Ile, TTT-Phe, ATA-Met, AAT-Asn and TAT-Tyr) are composed of T or A + T, whereas the least used codons (CTC-Leu, CTG-Leu, GCG-Ala, CGC-Ser and so on) have a high content of G + C. The relative synonymous codon usage exhibits extensive similarity with other mitogenomes from the same superfamily Sciaroidea (Wang et al. 2021). Codon usage of PCGs shows a significant bias of high A + T content, which plays a major role in the A + T bias of the entire mitogenome (Yang et al. 2013).

The 12S and 16S ribosomal RNA genes contain 824 and 1,361 bp, respectively. These genes are located between the genes for tRNA Phe and control region, and are separated by the gene for tRNA Val. The *B. impatiens* mitochondrial genome contains 22 tRNA genes (Table S1), which are interspersed along the genome, range in size from 47 to 72 nucleotides (Peng et al. 2006). Among them, 16 tRNA genes are located on the J-strand and six on the N-strand.

The phylogenetic tree was constructed based on all PCGs of seven published sciarid mitogenomes (Figure 1). It confirmed that *B*. *impatiens* belonged to the subfamily Megalosphyinae and is closely related to *B*. *amoena*. The topology of *Bradysia* species on the tree suggested the polyphyly of this genus, which was consistent with previous studies based on morphological characters and molecular markers (Menzel and Mohrig 2000; Shin et al. 2013). Species in the *B*. *tilicola* group are difficult to be differentiated morphologically, as they are usually distinguished only by the number of the apical megasetae on gonostylus (Sueyoshi and Yoshimatsu 2019). In addition, a single DNA marker is often insufficient to distinguish among species in such a closely related group. However, the mitogenome provides reliable evidence for validating these complex species concepts. The mitogenome data from this study also provides a better understanding for the phylogenetic relationship and speciation of *Bradysia*.

## Author contributions

**Yang Wang:** data curation; formal analysis; software; writing original draft; writing editing. **Caixia Liu:** writing - review & editing. **Qinyun Wang:** methodology; software; writing review & editing. **Hong Wu:** supervision. **Junhao Huang:** conceptualization, writing - review & editing, funding acquisition.

## Supplementary Material

Supplemental Material

## Data Availability

The genome sequence data that support the findings of this study are openly available in GenBank of NCBI at (https://www.ncbi.nlm.nih.gov/) under the accession no. MZ202360. The associated BioProject, SRA, and Bio-Sample numbers are PRJNA757190, SRR15573090, and SAMN20947072, respectively.
