# Supplementary material for: The complete mitochondrial genome of Bradysia impatiens (Diptera: Sciaridae)
Source: Mitochondrial DNA B Resour. 2022 Jun 20;7(6):1140–2. doi: 10.1080/23802359.2022.2080594 (PMC9225757; doi:10.1080/23802359.2022.2080594)
Supplement: Supplemental Material [file TMDN_A_2080594_SM4386.docx]

The complete mitochondrial genome of *Bradysia impatiens* (Diptera: Sciaridae)

Yang Wang^a^, Caixia Liu^a^, Qingyun Wang^a^, Hong Wu^a^, Junhao Huang^a^*

a National Joint Local Engineering Laboratory for High-Efficient Preparation of Biopesticide, Zhejiang A&F University, 666 Wusu street, Linan, Hangzhou, Zhejiang 311300, China

* Corresponding author: E-mail: huangjh@zafu.edu.cn, Tel: 86-571-63732758, Fax: 86-571-63740898

**Correspondence**

Junhao Huang, Zhejiang A&F University, 666 Wusu-street, Lin’an, Zhejiang 311300, China. E-mail: huangjh@zafu.edu.cn

**Table S1** Organization of the *Bradysia impatiens* mitochondrial genome.

| Gene/element | Abbreviation | Direction | Position | Size | Start | Stop |
| --- | --- | --- | --- | --- | --- | --- |
| A+T region (Control region) | Control region | F | 1-462 | 462 |  |  |
| tRNA^Phe^ | L | F | 463-527 | 65 |  |  |
| tRNA^Ile^ | I | F | 528-592 | 65 |  |  |
| tRNA^Gln^ | Q | R | 709-640 | 70 |  |  |
| tRNA^Met^ | M | F | 736-801 | 66 |  |  |
| NADH dehydrogenase subunit 2 | ND2 | F | 802-1842 | 1041 | ATG | TAA |
| tRNA^Trp^ | W | F | 1870-1935 | 66 |  |  |
| Cytochrome *c* oxidase subunit 1 | COI | F | 1941-3473 | 1533 | ATG | TAA |
| Cytochrome *c* oxidase subunit 2 | COII | F | 3586-4281 | 696 | ATA | TAA |
| tRNA^Lys^ | K | F | 4322-4393 | 72 |  |  |
| tRNA^Asp^ | D | F | 4407-4474 | 68 |  |  |
| ATP synthase F0 subunit 8 | ATP8 | F | 4484-4642 | 159 | ATA | TAA |
| ATP synthase F0 subunit 6 | ATP6 | F | 4636-5310 | 675 | ATG | TAA |
| Cytochrome *c* oxidase subunit 3 | COIII | F | 5324-6115 | 792 | ATA | TAA |
| tRNA^Gly^ | G | F | 6166-6233 | 68 |  |  |
| NADH dehydrogenase subunit 3 | ND3 | F | 6234-6587 | 354 | ATT | TAG |
| tRNA^Ala^ | A | F | 6630-6695 | 66 |  |  |
| tRNA^Tyr^ | Y | F | 6726-6788 | 63 |  |  |
| tRNA^Cys^ | C | F | 6798-6844 | 47 |  |  |
| tRNA^Ser^ | S | F | 6873-6934 | 62 |  |  |
| tRNA^Glu^ | E | F | 6979-7043 | 65 |  |  |
| tRNA^Phe^ | F | R | 7132-7064 | 69 |  |  |
| NADH dehydrogenase subunit 5 | ND5 | R | 8853-7132 | 1722 | AAT | CTA |
| tRNA^His^ | H | R | 8922-8854 | 69 |  |  |
| NADH dehydrogenase subunit 4 | ND4 | R | 10298-8961 | 1338 | CAT | TTA |
| NADH dehydrogenase subunit 4L | ND4L | R | 10597-10292 | 306 | AAT | TTA |
| tRNA^Thr^ | T | F | 10608-10674 | 67 |  |  |
| tRNA^Pro^ | P | R | 10750-10686 | 65 |  |  |
| NADH dehydrogenase subunit 6 | ND6 | F | 10751-11296 | 546 | ATA | TAA |
| tRNA^Arg^ | R | F | 11330-11394 | 65 |  |  |
| tRNA^Asn^ | N | F | 11410-11481 | 72 |  |  |
| Cytochrome *b* | cyt *b* | F | 11522-12640 | 1119 | ATA | TAA |
| tRNA^Ser^ | S | F | 12701-12765 | 65 |  |  |
| NADH dehydrogenase subunit 1 | ND1 | R | 13743-12793 | 951 | AAT | CTA |
| tRNA^Phe^ | L | R | 13811-13745 | 67 |  |  |
| 12S ribosomal RNA | 16S | R | 15195-13835 | 1361 |  |  |
| tRNA^Val^ | V | R | 15284-15217 | 68 |  |  |
| 16S ribosomal RNA | 12S | R | 16109-15286 | 824 |  |  |
| A+T region (Control region) | Control region | F | 16110-16479 | 370 |  |  |

**Table S2** Codon usage in the *Bradysia impatiens* mitochondrial protein-coding genes.

| **Amino acid** | **Codon** | **Number** | **Frequency** | **Condon usage (%)** |
| --- | --- | --- | --- | --- |
| Lys | AAA | 99 | 0.0265 | 89.2% |
|  | AAG | 12 | 0.0032 | 10.8% |
| Asn | AAC | 26 | 0.0070 | 12.2% |
|  | AAT | 187 | 0.0500 | 87.8% |
| Thr | ACA | 75 | 0.0201 | 46.6% |
|  | ACC | 8 | 0.0021 | 5.0% |
|  | ACG | 3 | 0.0008 | 1.9% |
|  | ACT | 75 | 0.0201 | 46.6% |
| Ser | AGA | 68 | 0.0182 | 19.6% |
|  | AGC | 5 | 0.0013 | 1.4% |
|  | AGG | 12 | 0.0032 | 3.5% |
|  | AGT | 29 | 0.0078 | 8.4% |
| Met | ATA | 283 | 0.0757 | 91.6% |
|  | ATG | 22 | 0.0059 | 7.1% |
|  | ATT | 4 | 0.0011 | 1.3% |
| Ile | ATC | 30 | 0.0080 | 7.7% |
|  | ATT | 358 | 0.0958 | 92.3% |
| Gln | CAA | 58 | 0.0155 | 90.6% |
|  | CAG | 6 | 0.0016 | 9.4% |
| His | CAC | 8 | 0.0021 | 11.9% |
|  | CAT | 59 | 0.0158 | 88.1% |
| Pro | CCA | 51 | 0.0136 | 40.8% |
|  | CCC | 9 | 0.0024 | 7.2% |
|  | CCG | 4 | 0.0011 | 3.2% |
|  | CCT | 61 | 0.0163 | 48.8% |
| Arg | CGA | 27 | 0.0072 | 54.0% |
|  | CGC | 3 | 0.0008 | 6.0% |
|  | CGG | 6 | 0.0016 | 12.0% |
|  | CGT | 14 | 0.0037 | 28.0% |
| Leu | CTA | 42 | 0.0112 | 7.1% |
|  | CTC | 1 | 0.0003 | 0.2% |
|  | CTG | 1 | 0.0003 | 0.2% |
|  | CTT | 33 | 0.0088 | 5.6% |
| Glu | GAA | 71 | 0.0190 | 93.4% |
|  | GAG | 5 | 0.0013 | 6.6% |
| Asp | GAC | 10 | 0.0027 | 15.9% |
|  | GAT | 53 | 0.0142 | 84.1% |
| Ala | GCA | 54 | 0.0145 | 40.0% |
|  | GCC | 15 | 0.0040 | 11.1% |
|  | GCG | 3 | 0.0008 | 2.2% |
|  | GCT | 63 | 0.0169 | 46.7% |
| Gly | GGA | 105 | 0.0281 | 52.2% |
|  | GGC | 7 | 0.0019 | 3.5% |
|  | GGG | 39 | 0.0104 | 19.4% |
|  | GGT | 50 | 0.0134 | 24.9% |
| Val | GTA | 78 | 0.0209 | 54.2% |
|  | GTC | 3 | 0.0008 | 2.1% |
|  | GTG | 5 | 0.0013 | 3.5% |
|  | GTT | 58 | 0.0155 | 40.3% |
| Stop | TAA | 10 | 0.0027 | 76.9% |
|  | TAG | 3 | 0.0008 | 23.1% |
| Tyr | TAC | 19 | 0.0051 | 11.4% |
|  | TAU | 147 | 0.0393 | 88.6% |
| Ser | TCA | 87 | 0.0233 | 25.1% |
|  | TCC | 7 | 0.0019 | 2.0% |
|  | TCG | 4 | 0.0011 | 1.2% |
|  | TCT | 135 | 0.0361 | 38.9% |
| Trp | TGA | 82 | 0.0219 | 84.5% |
|  | TGG | 15 | 0.0040 | 15.5% |
| Cys | TGC | 3 | 0.0008 | 7.9% |
|  | TGT | 35 | 0.0094 | 92.1% |
| Leu | TTA | 478 | 0.1279 | 80.9% |
|  | TTG | 36 | 0.0096 | 6.1% |
| Phe | TTC | 42 | 0.0112 | 11.1% |
|  | TTT | 336 | 0.0899 | 88.9% |


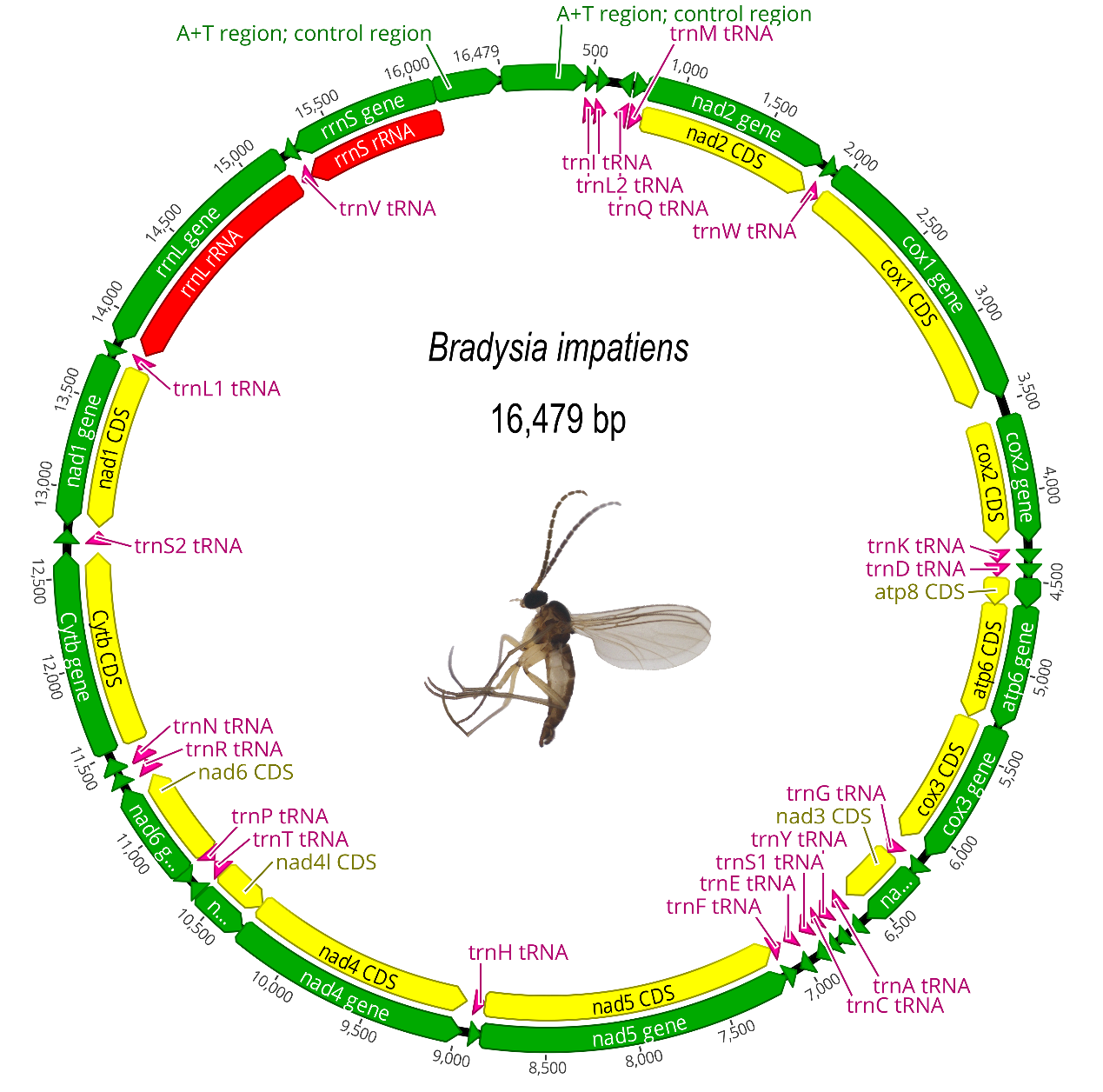


**Figure S1.** Map of the *Bradysia impatiens* mitogenome.
